# Correlation between mutation of MDR3 gene exon 6 and parenteral nutrition-associated cholestasis of preterm infants

**DOI:** 10.3892/etm.2014.1980

**Published:** 2014-09-19

**Authors:** XIU FANG YANG, GUO SHENG LIU, BING YI

**Affiliations:** 1 Department of Neonatology, The First Affiliated Hospital of Jinan University, Guangzhou, Guangdong 510630, P.R. China; 2 Department of Neonatology, Zhongshan People’s Hospital Affiliated to Sun Yat-sen University, Zhongshan, Guangdong 528403, P.R. China; 3 Zhongshan Cancer Institute, Zhongshan People’s Hospital Affiliated to Sun Yat-sen University, Zhongshan, Guangdong 528403, P.R. China

**Keywords:** premature infants, parenteral nutrition-associated cholestasis, MDR3 gene, gene mutation

## Abstract

The aim of this study was to investigate the association between the mutation of multidrug resistance 3 (MDR3) exon 6 and parenteral nutrition-associated cholestasis (PNAC) in preterm infants. A total of 41 preterm infants with PNAC formed the experimental group, and 56 preterm infants receiving total parenteral nutrition (TPN) for >14 days but without cholestasis formed the control group. Genomic DNA was extracted from peripheral venous blood leukocytes. Polymerase chain reaction was used to amplify exon 6 of the MDR3 gene. The target band of MDR3 gene exon 6 was identified in all blood samples from all cases. We identified five cases with C. 504 C>T heterozygous mutations of exon 6 of the MDR3 gene and 14 cases with C. 504 C>T homozygous mutations in the experimental group. In the control group, we identified seven cases with the C. 504 C>T homozygous mutation and six cases with the C. 504 C>T heterozygous mutation. The distribution of the T/C allele frequency of C. 504 in exon 6 of the MDR3 gene between the experimental group and control group was statistically significant (P<0.05). Further analysis revealed the odds ratio of the T/C allele frequency of the C. 504 mutation in exon 6 of the MDR3 gene between the experimental group and control group to be 0.316. Point mutation C. 485 T>A was detected in one case in the experimental group. The C. 504 C>T and C. 485 T>A MDR3 mutations in exon 6 are possibly responsible for the development of PNAC in infants. C. 504 C>T may not be the only risk factor of neonatal PNAC. In order to further confirm the association between exon 6 of the MDR3 gene and PNAC, a large-sample multicenter study should be carried out.

## Introduction

Premature infants with gastrointestinal intolerance require total parenteral nutrition (TPN) to enable metabolic growth. In recent years, with the development of medicine, a greater proportion of extremely low birth weight infants and very low birth weight infants are surviving than before due to the application of parenteral nutrition. However, certain complications may occur in premature infants receiving TPN, due to their specific physiological and pathological characteristics. The complications in premature infants receiving TPN include sepsis, thromboembolism, metabolic imbalance and hepatobiliary complications. Parenteral nutrition-associated cholestasis (PNAC) has received the most attention due to the liver damage sustained (1).

PNAC is defined, based on previously established definitions, as a direct bilirubin level of >2.0 mg/dl following a prolonged course of TPN (>2 weeks) and when other causes, including surgical and metabolic diseases, have been ruled out. Severe PNAC was defined as a direct bilirubin level of >5.0 mg/dl. Research has revealed that in premature infants, particularly those with very low birth weights, the incidence of PNAC is as high as 50% (2). The pathogenesis of PNAC remains unclear. The histological features of PNAC are intracellular and intracanalicular cholestasis, steatosis and periportal inflammation, and eventually fibrosis. Previous studies have suggested that PNAC occurs in premature infants due to premature liver, imperfect bile acid metabolism function and liver uptake ability, disorder of bilirubin enterohepatic circulation, immature gastrointestinal mucosal barrier function and immune system development, lack of fasting gastrointestinal nutrition stimulation, infection, TPN solution imbalance and parenteral nutrition toxic ingredients (3–5). The above factors may cause liver damage and the abnormal metabolism of bile in the liver. PNAC may be reversible in its early stage. However, if is not prevented as early as possible, PNAC may develop into cholestasis, liver fibrosis, other irreversible liver damage, liver failure and even mortality. It is therefore necessary to explore the pathogenesis of PNAC.

Intestinal fat digestion requires adequate lipase activity and bile acid concentrations, both of which are low in preterm infants. Bile acids are required for the emulsification of fat before and during lipolysis, and they act as an activator for bile acid-dependent lipase in breast milk. Stable solubilization of fat during digestion by bile acids is one of the main prerequisites for sufficient fat absorption (6).

The etiology of PNAC has been considered to be multifactorial and the main contributory factor in each case is often unclear. In addition to apparent risk factors, including early exposure to TPN and total caloric overloading, the genetic factors for PNAC are of significant interest (7). The present study aimed to analyze the association between the mutation of multidrug resistance 3 (MDR3) gene exon 6 and PNAC. The MDR3 gene encodes the MDR3 protein. MDR3 gene mutation may lead to a decrease in the amount of MDR3 protein or MDR3 protein dysfunction, which may cause a lack of phospholipids in bile, an increase in bile stone formation, or further small bile duct obstruction and cholestasis (8,9). With the development of genomics, increasing evidence is emerging to demonstrate that genetic factors, including mutation of the MDR3 gene, play a significant role in the pathogenesis of intrahepatic cholestasis (10–12). The current study set out to explore the pathogenesis of PNAC in preterm infants by researching the correlation between MDR3 gene exon 6 and PNAC in premature infants.

## Patients and methods

### Patients

This study enrolled 41 preterm infants with PNAC and 56 preterm infants receiving TPN for >14 days but without PNAC who were admitted to the neonatal intensive care unit and sick neonatal wards of Zhongshan People’s Hospital Affiliated to Sun Yat-sen University (Zhongshan, China), between June 1, 2011 and December 15, 2013. The preterm infants with PNAC formed the experimental group, and the preterm infants without PNAC formed the control group. The study was conducted in accordance with the declaration of Helsinki and with the approval of the Ethics Committee of Zhongshan People’s Hospital (Zhongshan, China). Written informed consent was obtained from the parents/guardians of all participants.

The criteria for diagnosis of PNAC (13) were as follows: i) parenteral nutrition >14 days; ii) jaundice, hepatosplenomegaly, discolored stools and elevated liver enzymes; (iii) direct bilirubin level of >2 mg/dl, and/or ratio of direct bilirubin and total bilirubin of >20%; (iv) without virus infection (hepatitis A, B, C and cytomegalovirus); (v) exclusion of certain causes of neonatal cholestasis including biliary atresia, choledochal cyst, bile duct dilatation, bump oppressive and aneurysm surgery disease and metabolic diseases by methods including abdominal ultrasound, magnetic resonance holangiopancreatography and metabolic screening.

### Laboratory examinations

Cytomegalovirus, *Toxoplasma gondii* and syphilis blood culture were routinely checked, while C-reactive protein (CRP), blood glucose and liver function [alanine transaminase (ALT), aspartate aminotransferase (AST), γ-glutamyl transpeptidase (r-GGT), total bilirubin, direct bilirubin and total bile acid] tests were performed weekly. Genomic DNA was extracted from peripheral venous blood leukocytes of all cases. The samples were stored in an ultra-low temperature freezer at −80°C.

### Amplification of MDR3 exon 6 by polymerase chain reaction (PCR)

Genomic DNA was extracted from the white blood cells using the phenol-chloroform method (14). PCR-restriction fragment length polymorphism was used to synthesize primers. The primer was designed with Primer 5 software (15). The DNA concentration was quantified by spectrophotometry using a UV-5200 UV/VIS spectrophotometer (Shanghai Metash Instruments Co. Ltd, Shanghai, China). The primers for MDR3 exon 6 were as follows: Forward, 5′-GGGGGTGGTGGCTCATGCTATA-3′ and reverse, 5′-GGGGGAAAGCCAACATGCAAT-3′. The primary PCR product fragments were 720 bp. PCR was used to amplify exon 6 of the MDR3 gene, and was generally performed in a reaction volume of 50 μl with 100 ng genomic DNA, 45 μl Platinum^®^ PCR SuperMix (Invitrogen Corporation, Grand Island, NY, USA), 200 μmol/l deoxynucleoside-5-triphosphate (Takara, Dalian, China) and 20 μmol/l of each primer. PCR conditions included an initial denaturation step at 94°C for 3 min, followed by 30 cycles of denaturation at 94°C for 15 sec, and annealing at 72°C for 30 sec. The PCR reaction was terminated after an extension step at 72°C for 5 min. PCR products (3 μl) were observed following electrophoresis of 1% agarose gel (Bio-Rad, Hercules, CA, USA) with an automatic gel imaging system and a DNA marker (Takara). A clear, bright band was present indiciating successful gel electrophoresis. Next, amplification of the MDR3 gene DNA was carried out by PCR (Bio-Rad) and the mutation of exon 6 was detected in the MDR3 gene through the restriction enzyme (sex AI) digestion method, using an ABI Prism^®^ 3100 sequencer (Applied Biosystems, Foster City, CA, USA). In the DNA sequence analysis, DNA sequences were compared with the human genome sequence of the MDR3 gene in GenBank (16).

### Statistical analysis

Statistical analysis was performed using SPSS 17.0 software (SPSS, Inc, Chicago, IL, USA). The genotype frequency and allele frequency distribution of the two groups were compared with the χ^2^ test. The data from the two groups were compared with the Student’s t-test, and P<0.05 was considered to indicate a statistically significant difference. Gene mutations were analyzed with BioEdit protein contrast software (17).

## Results

### Clinical data

The clinical data comparison between the experimental group and control group revealed that the differences in gender, hypoxia, sepsis and hemolytic disease between the two groups were not statistically significant (P>0.05 for all), and that the differences in gestational age and intravenous nutrition time were statistically significant between the two groups (P<0.05 for both). The gestational ages in the experimental group were lower than those in the control group. The duration of TPN in the experimental group was longer than that in the control group (Table I).

### Electrophoresis results

**Figure 1:**
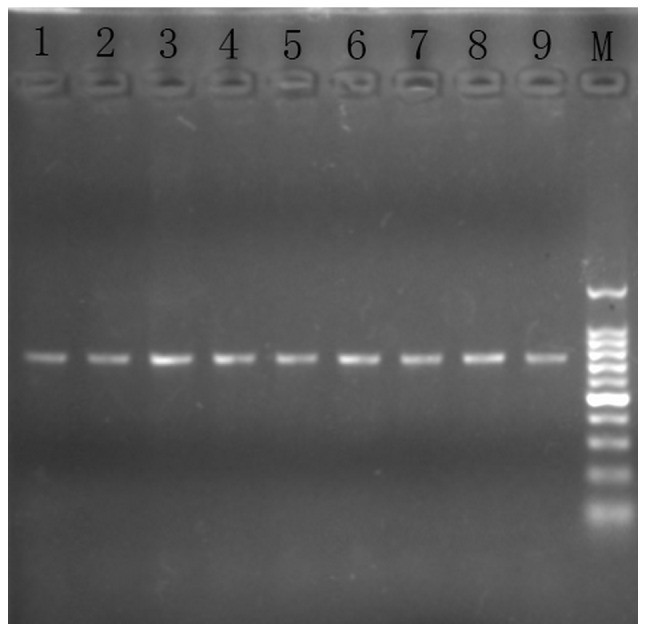
Amplified fragments of multidrug resistance 3 gene exon 6 (720 bp). M, DNA marker DL2000.

The electrophoresis results revealed a clear band of MDR3 gene exon 6 amplified fragments. The size was 720 bp (Fig. 1).

### DNA sequencing

**Figure 2:**
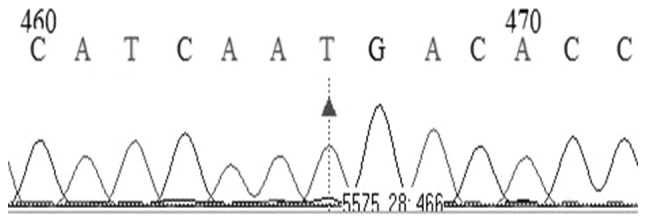
DNA sequencing of synonymous mutation in multidrug resistance 3 gene exon 6 (19 cases). Mutation position, ATCAATGAC; nucleic acid position, C. 504 C>T; protein position, 168 aspartic acid synonymous mutation (AAC168AAT).

The DNA sequence analysis in exon 6 of the MDR3 gene revealed a single nucleotide substitution, a C. 504 C>T homozygous mutation in 14/41 premature infants and a C. 504 C>T heterozygous mutation in 5/41 premature infants in the experimental group. It also revealed a C. 504 C>T homozygous mutation in 7/56 premature infants and a C. 504 C>T heterozygous mutation in 6/56 premature infants in the control group (Fig. 2). This was a silent mutation at the protein level: P. Asn 168 Asn. The distribution of T/C allele frequencies (χ^2^ and logistics regression analysis, SPSS 17.0) of C. 504 C>T was significantly different between the experimental group and the control group (P=0.001; OR=3.098; 95% confidence interval, 1.610–5.952; Table II). The distribution of CC, TT and CT genotype frequencies was statistically significant between the experimental group and the control group (P<0.05; Table III).

**Figure 3:**
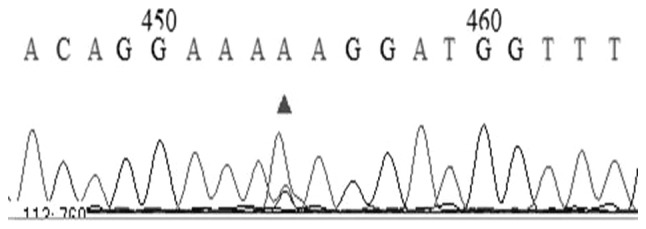
DNA sequencing of missense mutation in multidrug resistance 3 gene exon 6 (one case). Mutation position, GAAAAAGGA; nucleic acid position, C. 485T>A; protein position, P. Ile 162 Lys, missense mutation.

In addition, DNA sequencing revealed a further single nucleotide substitution in exon 6: C. 485 T>A (P. Ile 162 Lys) in 1/41 premature infants in the experimental group, which was a missense mutation (Fig. 3).

### Laboratory results

Laboratory results revealed that there were no statistically significant differences in the level of ALT, total bilirubin and direct bilirubin between the premature infants with the C. 504 C>T mutation and the premature infants without the C. 504 C>T mutation in the experimental group (P>0.05 for all). There was a statistically significant difference in the level of total bile acid and r-GGT between the preterm infants with the C. 504 C>T mutation and the preterm infants without the C. 504 C>T mutation (P<0.05 for both). The results revealed that the patients with C. 504 C>T in the experimental group had raised serum r-GGT (Table IV). Table V shows that the preterm infant with the C. 485 T>A mutation had raised r-GGT.

## Discussion

As the development of treatment technology improves and the application of TPN is increased, the survival rate of premature infants increases significantly (18). However, the complications of parenteral nutrition, including PNAC, have become a significant problem which neonatologists need to address. Severe cases may lead to malnutrition, biliary cirrhosis, liver failure and even mortality. Research has revealed that low birth weight infants with severe PNAC are prone to severe rickets (19). With the development of genomics, there is increasing evidence demonstrating that genetic factors, including mutation of the MDR3 gene, play a significant role in the pathogenesis of intrahepatic cholestasis (20–25).

The MDR3 gene is also known as the ATP binding cassette B4 gene, which belongs to the ATP binding transporter (ATP binding cassette, ABC) members of the supergene family of glycoprotein genes. The MDR3 gene encodes the MDR3 protein. The MDR3 gene is divided into four regions: Two homologous nucleotide binding domains (NBDs) towards the cytoplasm, which can combine hydrolysis of ATP and which have a highly conserved sequence in the ABC family; and two homologous transmembrane regions consisting of six hydrophobic segments, which transfer substrate through multiple transmembrane and export channels. The MRD3 gene is located in 7q21.1. The span is 74 kb, and there are 4.1 kb in CDNA. It contains 28 exons, including 27 exons with the coding sequence. The two nucleotides of the MDR3 gene encoding the protein zone (NBD) are encoded by five exons (exons 12, 14, and exon 25, 26, 27). Each of the 12 transmembrane segments is coded by one separate exon, and the other four transmembrane segments are encoded by two exons (26,27).

Studies have shown that MDR3 participates in the secretion of bile lecithin as the liver bile capillary membrane transporter (28–30). Phospholipids in bile are mainly phosphatidylcholine. Phosphatidylcholine can be emulsified by bile salt and cholesterol. It prevents cholesterol precipitation and protects the bile duct epithelium from bile salt injury. The expression of the MDR3 gene may affect the concentration of bile lecithin. Reduced expression of MDR3 leads to a reduction in the secretion of lecithin and elevated vesicle cholesterol content, and may also lead to bile duct damage, gallstone deposition, inflammation and further biliary liver lesions (31,32).

Several studies have suggested that progressive familial intrahepatic cholestasis, cholelithiasis, pregnancy associated with intrahepatic cholestasis and drug-induced cholestasis are closely correlated with the MDR3 gene (22,33,34). Correlation between MDR3 gene mutation and PNAC in premature infants will become a prominent research topic. Studies have demonstrated that exon 6 of MDR3 gene mutations is associated with cholestasis (35,36). The aim of the present study was to investigate the correlation between exon 6 of MDR3 and premature infants with PNAC.

The present study revealed that there was a correlation between the MDR3 gene exon 6 C. 504 C>T synonymous mutation and PNAC in premature infants. In premature infants with PNAC, the serum r-GGT and total bile acid levels in the cases with the C. 504 C>T mutation were higher than those in the cases without the mutation. One case in the experimental group was detected with a C. 485 T>A missense mutation and raised r-GGT. Our results demonstrate that there is a close correlation between mutation of MDR3 exon 6 and cholestasis.

The results reveal that C. 504 C>T and C. 485 T>A MDR3 mutations in exon 6 were detected in premature infants with PNAC. These mutations may be responsible for the development of PNAC in infants. The C. 504 C>T mutation in exon 6 of the MDR3 gene may be an individual risk factor of PNAC. It is necessary to study all the exons in the MDR3 gene further and increase the sample size in order to reveal the correlation between the MDR3 gene and PNAC.

## Figures and Tables

**Table I:** Clinical data of the experimental group and control group.

Variables	Experimental group	Control group	χ^2^/t	P-value
Cases	41	56	-	-
Male (n)	23	25	1.242	>0.05
Gestation (d)	212±9	224±12	6.042	<0.05
Duration of PN (d)	50±5	42±8	5.629	<0.05
Anoxia (n)	9	11	0.077	>0.05
Septicemia (n)	6	9	0.037	>0.05
Hemolysis (n)	7	6	0.825	>0.05

PN, parenteral nutrition.

**Table II:** Comparison of the distribution of genotype frequencies in C. 504 C>T in the two groups.

SNPs	Genotype	Experimental group	Control group	χ^2^	P-value
C. 504 C>T	CC	22 (53.7%)	43 (76.8%)	7.058	0.029
	CT	5 (12.2%)	6 (10.7%)		
	TT	14 (34.1%)	7 (12.5%)		

**Table III:** Comparison of the distribution of allele frequencies in C. 504 C>T in the two groups.

SNPs	Allele	Experimental group	Control group	OR	95% confidence interval	χ^2^	P-value
C. 504 C>T	C	49 (59.8%)	92 (82.1%)	0.316	0.164–0.609	12.357	0.001
	T	33 (40.2%)	20 (17.9%)				

OR, odds ratio.

**Table IV:** Comparison of laboratory results between infants with and without C. 504 C>T mutation.

Groups	Cases	ALT (U/l)	T-BIL (μmol/l)	D-BIL (μmol/l)	TBA (μmol/l)	r-GGT (U/l)
With C. 504 C>T mutation	19	127±20.1	128.8±20.7	70.2±10.8	68.9±11.4	78±7.2
Without C. 504 C>T mutation	22	118±19.3	120.4±23.9	69.4±9.4	67.9±9.3	41±5.1
t		1.456	1.206	0.251	0.303	19.201
P-value		>0.05	>0.05	>0.05	>0.05	<0.05

ALT, alanine transaminase; T-BIL, total bilirubin; D-BIL, direct bilirubin; TBA, total bile acid; r-GGT, γ-glutamyl transpeptidase.

**Table V:** Laboratory results of infant with C. 485 T>A mutation.

Case	Number	ALT (U/l)	T-BIL (μmol/l)	D-BIL (μmol/l)	TBA (μmol/l)	r-GGT (U/l)
C. 485 T>A mutation	1	140	150.3	80.2	90.6	116

ALT, alanine transaminase; T-BIL, total bilirubin; D-BIL, direct bilirubin; TBA, total bile acid; r-GGT, γ-glutamyl transpeptidase.
